# Warm‐night temperature alters paternal allocation strategy in a North temperate‐zone butterfly

**DOI:** 10.1002/ece3.8120

**Published:** 2021-11-23

**Authors:** Elena Rosa, Marjo Saastamoinen

**Affiliations:** ^1^ Organismal and Evolutionary Biology Research Programme University of Helsinki Helsinki Finland; ^2^ Helsinki Institute of Life Science University of Helsinki Helsinki Finland

**Keywords:** behavior, climate change, drought stress, oviposition preference, resource allocation

## Abstract

Warming temperatures are greatly impacting wild organisms across the globe. Some of the negative impacts of climate change can be mitigated behaviorally, for example, by changes in habitat and oviposition site choice. Temperatures are reportedly warming faster at night than during the day, yet studies assessing the impacts of increasing night temperature are rare. We used the Finnish Glanville fritillary butterfly (*Melitaea cinxia*) as study species and exposed adult butterflies of both sexes to warmer night conditions. Under a seminatural outdoor enclosure, we assessed whether females base their oviposition choices primarily on habitat site characteristics (open, suggestive of dry meadows, versus covered by a coarse canopy, suggestive of pastures) or on plant condition (dry vs. lush), and if their choice is altered by the thermal conditions experienced at night. As exposure to warmer environmental conditions is expected to increase resting metabolic rate and potentially reduce life expectancy, we further assessed the fitness implications of warm‐night temperatures. We found that females prefer open sites for oviposition and that females do not switch their oviposition strategy based on the thermal conditions they experienced at night prior to the reproductive event. Exposure to warm nights did not influence female lifespan, but the egg hatching success of their offspring was reduced. In addition, we found that males exposed to warm nights sired larger clutches with higher hatching rate. As warm‐night exposure reduced male lifespan, this may imply a switch in male resource allocation strategy toward increased offspring quality. The present work adds on to the complex implications of climate warming and highlights the importance of the often‐neglected role of males in shaping offspring performance.

## Graphical abstract

We tested the impact of nights warmer than usual on female oviposition site preference and on parental resource allocation strategy. We found that females did not switch their oviposition preference to sites less likely to undergo severe drought, despite experiencing warm‐night temperatures. Interestingly, warm‐exposed males improved the performance of their offspring by presumably switching their resource allocation strategy.

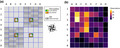

## 1 INTRODUCTION

Climate change is gradually increasing the average global temperature (Van Vuuren et al., 2008), and it has been estimated that temperatures are warming faster at night than during daytime (Cox et al., 2020). Rising temperatures have been shown to impact local populations in many ways, for instance by causing changes in behavior, dispersal, development, and phenology among others (Root et al., 2003). Some examples of the negative impacts of climate change include the alteration in the occurrence of sexually selected features (Spottiswoode et al., 2006), sex ratio shifts in species where sex is determined thermally (Santidrián Tomillo et al., 2015), the modification of the timing of foraging and breeding of migratory species (Møller et al., 2008), and phenological mismatches between interacting species (Menzel et al., 2006; Ovaskainen et al., 2013; Walther, 2010). Plastic responses and behavioral modifications are however expected to take place to mitigate, or at least cope with, some of the effects of climate change (Beever et al., 2017; Kearney et al., 2009; Refsnider & Janzen, 2012). However, the impacts of rising night temperature on life history responses and their potential behavioral implications have been largely neglected in research.

Impacts of climate change may be especially pronounced on the sensitive and sessile developmental stages, such as the early developmental stages in insects, and these effects may be especially pronounced in species lacking direct parental care (Nussbaum & Schultz, 1989; reviewed in Refsnider & Janzen, 2010). Apart from cases where the conditions experienced by the newly produced eggs cannot be predicted by the parents (such as species producing pelagic eggs, e.g., Röhrs et al., 2014), these early‐life conditions are largely determined by maternal oviposition site choices, as commonly seen in insect herbivores (Albanese et al., 2008; Gotthard et al., 2004; Rausher, 1979; Tjørnløv et al., 2015), reptiles (Brown & Shine, 2004; Kolbe & Janzen, 2001; Mitchell et al., 2013; Shine et al., 1997), and some birds (Lloyd & Martin, 2004). The conditions experienced at the oviposition sites, such as temperature, resource abundance, and predation risk (Refsnider & Janzen, 2010; Scheirs & De Bruyn, 2002; Thompson, 1988), and the spatial distribution of the sites (Friberg et al., 2008; Friberg & Wiklund, 2019; Wiklund & Friberg, 2008) are key determinants of oviposition choice. However, it is unknown whether the abiotic conditions the females themselves experience prior the reproductive event impact their oviposition choice. It could be, for example, beneficial for females to switch their behavior and lay eggs in more shaded areas, if they were exposed to very warm temperatures prior the oviposition. Previous work shows that at least suddenly changing environmental conditions force females to rapidly switch their ovipositing choices (e.g., Roitberg et al., 1993).

Offspring performance and success are also determined by the amount of resources parents can allocate to them at the moment of reproduction. This parental resource allocation can also be influenced by climate change. In ectothermic organisms, rising temperatures are expected to increase metabolic rate (Dillon et al., 2010) and consequently accelerate the pace of life, causing a shorter lifespan or an earlier death (Pearl, 1928). This is expected to alter the timing of reproductive efforts and shift resource allocation from future to current reproduction (reviewed in Metcalfe & Monaghan, 2001). It is possible that warming conditions cause adults to dissipate resources more rapidly and simply result in negative consequences for the offspring. However, warmer conditions may also result into more effective use of resources and hence improve offspring performance.

In this study, we were specifically interested in the effects of warmer than average nights on the behavior and life history of an ecological model species occurring in the North temperate zone, the Finnish Glanville fritillary butterfly (*Melitaea cinxia*). In recent years, the local butterfly population has faced warmer summers and extreme drought events (Kahilainen et al., 2018; van Bergen et al., 2020) placing our work in an ecologically relevant context. Even though these butterflies are known to have higher preference for “open microhabitats” (Salgado et al., 2020), this preference may become a risky strategy in future and lead to local population crashes (van Bergen et al., 2020). More specifically, we were interested in whether warmer nights caused a shift in maternal behavior in terms of oviposition site and plant preference. We predicted that such shift may take place as it could ensure better microclimatic conditions for their offspring under warmer climates. We also tested whether the night conditions experienced by the parents, both females and males, had any influence on fitness‐related traits including offspring hatching success. We expected changes in resource allocation strategies as a consequence of potentially increased metabolic rate resulting in accelerated pace of life under warmer night conditions. Previous work showed that a 10℃ raise in temperature can induce a 2‐ to 3‐fold increase in resting metabolic rate in this species (Niitepõld, 2010). Notably, we addressed the role of both sexes, as often times the impact of paternal contribution to offspring fitness is neglected.

## 2 MATERIALS AND METHODS

### 2.1 Study species

The individuals used (*N* = 163) were a laboratory F3 generation of wild‐collected individuals from the Åland Islands (Ojanen et al., 2013). The larvae were reared under laboratory conditions (28:15°C, L:D, 12:12 hr) on control *Plantago lanceolata* cuttings (i.e., following the same conditions as those denoted here as “lush”—see below). Individuals were weighed upon pupation, as pupal mass is a good proxy of adult mass (Rosa & Saastamoinen, 2017). To obtain synchronized adult eclosion upon release in an outdoor seminatural setup (see below), pupae were kept at 12:8°C, L:D, 12:12 hr for 2–17 days, for maximum 2 days in a row.

### 2.2 Butterfly overnight thermal treatment

**FIGURE 1:**
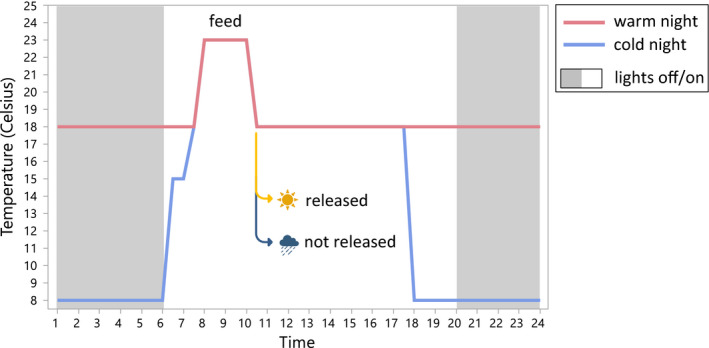
Scheme of daily temperature and lighting cycles for warm and control night treatments. Every morning at 7 the temperature was increased up to 23°C so that between 8 and 10 a.m. butterflies were active enough to feed on a 20% honey:water solution. If the outdoor conditions were sunny and warm (above 18°C) enough for the butterflies to be active, they were released after feeding. Otherwise, the temperature dropped again to 18°C and the butterflies were left in the SANYO cabinets. Humidity was constant in all the chambers

We wanted to test the effect of warming conditions on life history and behavioral traits by exposing half of the butterflies to a warm‐night treatment, which replicated the warmest night temperatures recorded on thermal peak years in the Åland Islands. The warm‐night treatment was initiated with 24‐hr‐old adults on the night before their release in the caged outdoor environment (Figure 1). Adults were individually marked and randomly placed to warm (18°C) or control night rearing temperatures (8°C; see detailed cycles in Figure 1). During the day, butterfly activities were closely monitored within an outdoor population enclosure (see Section 2.3), where butterflies were released each morning and then recollected in the evening to be returned to the respective overnight thermal treatments. On days when the weather was unsuitable for the butterflies to be active (i.e., raining), they were all kept indoors at 18°C (Figure 1).

### 2.3 Plant treatments and oviposition sites setup

**FIGURE 2:**
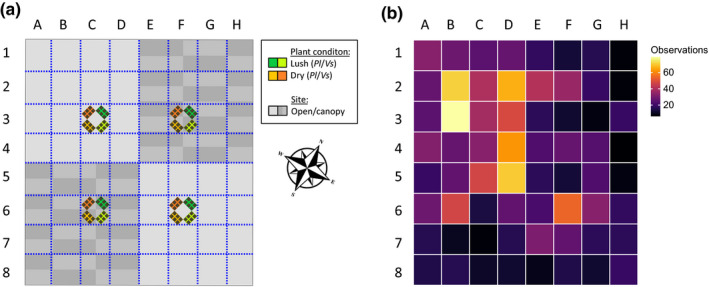
Map of the outdoor butterfly enclosure (32 × 26 × 3 m) showing the location of shading nets and oviposition plants (a), and heatmap displaying the number of observations in each grid plot during the experiment (b). Blue dotted lines represent the grid the enclosure is divided into allowing the observers to locate the activities of the butterflies during the day. Transects are performed starting from cell A1‐A8 → B8‐B1 and so on. Each cell represents a rectangle of 4 × 3.25 m

Ninety‐six fully developed host plants, 48 *Veronica spicata,* and 48 *P*. *lanceolata* were used as oviposition plants in the experiment. These plants were equally divided between two watering treatments: “dry” and “lush.” For the 10 days preceding the experiment and during the experiment, the plants were watered every second day with 40 ml for the “dry” treatment, and ad libitum for the “lush” treatment. The “lush” treatment resulted in plants that had only green leaves all the time, and thus, resembled plants found in pastures and shadier parts of outcrop meadows (Ojanen et al., 2013). The plants were further divided among four sectors within the outdoor cage (Figure 2a), so that each sector hosted 6 plants per species and treatment (i.e., 24 plants per sector). Two of these sectors were covered with a shading mesh, mimicking a coarse canopy habitat that may be found in pastures near forest margins, and the other two were open sites in full sun mimicking dry meadows (Figure 2a). Plants were randomized daily within and among sectors. One data logger for temperature and humidity (i.e., four in total) was placed at each of the oviposition sites at about 10 cm from the ground level, which is the height at which butterflies oviposited on potted plants. Temperature and humidity records were collected only during the times of the day when butterflies were in the enclosure (i.e., between 10 a.m. and 6 p.m.). Differences in lighting between open and canopy‐covered areas were assessed with one data logger per condition over two days at the end of the experiment.

### 2.4 Observations under seminatural conditions

Every morning at 10 a.m. adults were released in the central part of the 32 × 26 × 3 m outdoor population enclosure (e.g., Rosa & Saastamoinen, 2017), where the butterflies were monitored. Matings, ovipositions, and mortality inside the cage were recorded during three daily transects (11a.m., 1, and 3p.m.), whereby one observer recorded all the butterflies he/she encountered as well as the activities they were performing (basking, flying, mating, ovipositing; Figure 2). The recording of synchronous mating and oviposition events was possible because there were up to five people in the enclosure, and always at least one person in proximity the oviposition sites. Hence, oviposition sites were monitored constantly. Plant choice and oviposition time were recorded, oviposition duration was timed, and after each oviposition, the eggs were collected. Every day after 4 p.m., once the butterflies became inactive, as many butterflies as possible (i.e., at least 80% of the butterflies released in the cage in the morning) were recollected within a 2‐hr period (Appendix S2). The butterflies were brought indoors in the assigned warm/control night temperature treatment, which was implemented throughout the adult life. The following morning the butterflies were fed and then again re‐released into the enclosure. The collected eggs were brought into the laboratory, counted, and reared in controlled conditions (28:15°C, L:D, 12:12 hr) to quantify hatching rate. Each egg cluster was carefully disassembled with a fine paint brush to allow counting of individual eggs and then reassembled to minimize egg mortality due to desiccation. Once the eggs hatched, approximately 10 days later, the emerged larvae were counted also with the aid of a fine paint brush. Because eggs are normally fathered by the last mate (Sarhan & Kokko, 2007), paternity of most clutches could be traced from the mating records.

### 2.5 Statistical analysis

All data were analyzed with R for Windows (v. 3.6.1; R Core Team, 2019). Preference for plant species (*P*. *lanceolata* vs. *V. spicata*), condition (lush vs. dry), and habitat site (open vs. canopy) was analyzed with a chi‐square goodness‐of‐fit test. The remaining variables were analyzed with a mixed‐model approach using the package *lmerTest* (Kuznetsova et al., 2017). Because females lay several egg clutches in their lifetime, we tested the effect of night temperature treatment on female oviposition plant and site choice with a mixed‐model approach for repeated measures, using female ID as random factor and clutch rank as a covariate (Bates et al., 2015). The response variables clutch size, time of the day when an oviposition was initiated, and oviposition duration were tested with a mixed model including female and male night temperature treatment, plant species and watering condition, site, and clutch rank as fixed factors and female ID as random factor. The best model was selected based on the lowest AIC value with ΔAIC > 4 as threshold. When the ΔAIC was not met, model averaging was performed using the *MuMIn* R package (Barton, 2020). General fitness parameters as likelihood to mate and to sire offspring, number of clutches and total eggs produced, total hatching success, and lifespan were tested separately by sex using night temperature treatment, pupal mass and their interaction as fixed factors, and family of origin as random factor.

## 3 RESULTS

**FIGURE 3:**
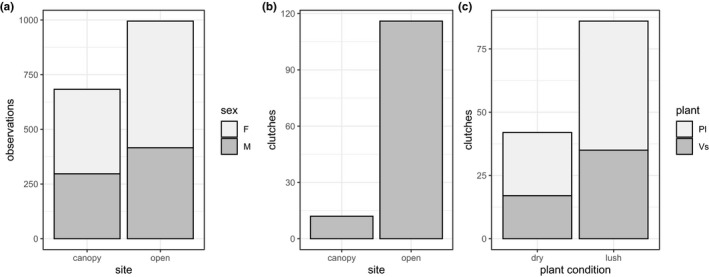
Butterfly choosiness in terms of (a) number of adult observations in open versus canopy sites, clutches found in (b) open versus canopy sites, and (c) on dry versus lush *P. lanceolata* or *V. spicata*. Females (“F”) and males (“M”) in (a) and *P. lanceolata* (“Pl”) and *V. spicata* (“Vs”) in (c) are represented by light and dark gray fill, respectively

There was no difference in the recollection success between butterflies assigned to the different night temperature treatments (*p* > .6 in males and *p* > .8 in females). Open sites were on average warmer, more luminous, and less humid than canopy‐covered ones (Table 1). Sixty‐six of 86 females mated, and 34 females laid eggs during the experiment. During the daily monitoring, only one out of 128 oviposition events was missed, while about 20% of the mating pairs were missed. The mating frequency of females was 1.22; hence, the vast majority of them mated only once. Adults showed a clear preference for open sites both in terms of number of observations (*χ* ^2^ = 58.1, *p* < .0001, Figures 2b and 3a, Table 2) and female ovipositions (*χ* ^2^ = 84.5, *p* < .0001, Table 2, Figure 3b). Ovipositions in shaded sites were prevalently on days ~1°C warmer and ~1% less humid than average (Table 1). Oviposition time was unaffected by the variables tested (Table S3). Oviposition duration lasted on average 38 ± 2.6 min, and in general, ovipositions lasted longer in shaded sites (*z* = 2.11, *SE* = 9.5, *p* = .04, Table S3). Females had a clear preference to oviposit on lush plants (*χ* ^2^ = 15.125, *df* = 1, *p* = .0001, Table 2, Figure 3c) and on *P. lanceolata* (*χ* ^2^ = 4.5, *df* = 1, *p* = .03, Table 2, Figure 3c). Night temperature treatment of the female had no impact on her oviposition choice (*p* > .3 for all, Table S1). The temperature treatment of the female's mating partner, on the other hand, influenced female oviposition plant choice, as females that mated with warm‐exposed males had an even stronger preference for *P. lanceolata* as oviposition host plant (*χ* ^2^ = 7.9, *p* = .005, Table S1).

**TABLE 1:** Mean temperature and relative humidity (RH) averages measured during the specific times of egg laying at the selected sites, and at all sites

	Open	Canopy	Significance (*p*‐value)
Temp. at oviposition site (°C)	34.23 ± 0.41	30.28 ± 1.08	.022*
Temp. at all sites (°C)	31.89 ± 0.35	32.94 ± 1.14	.27
RH at oviposition site (%)	41.85 ± 0.60	47.00 ± 2.38	.031*
RH at all sites (%)	44.41 ± 0.55	43.33 ± 1.74	.55
Luminosity (lx)	46,756.08 ± 1,415.79	20,129.38 ± 305.44	<.0001***

Note

Measures “at oviposition site” are real‐time measurements at the actual site where the oviposition was recorded. Measures “at all sites” are averages of all possible oviposition sites during the times oviposition took place. This comparison was made to assess differences between chosen and nonchosen sites (i.e., a particularly warm and dry days). Luminosity was measured over two days at the end of the experiment. Values are given as means ± standard error.

**TABLE 2:** Number of observations (both sexes) during the transects in the different sites, as well as number of ovipositions (i.e., only females) on different plant species, watering condition, and habitat sites. *χ* ^2^ statistics is reported for all the listed conditions

	*N*	*df*	*χ* ^2^ value	*χ* ^2^ *p*‐value
Observations				
Open	995	1	58.0	<.0001
Canopy	683			
Ovipositions				
*Plantago lanceolata*	76	1	4.5	.034
*Veronica spicata*	52			
Dry	42	1	15.1	.001
Lush	86			
Open	117	1	87.8	<.0001
Canopy	11			

**FIGURE 4:**
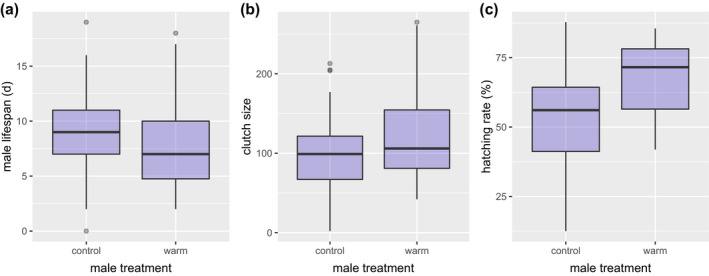
The impact of male warm‐night treatment on (a) his lifespan, (b) the size of the clutches he sired, and (c) their hatching percentage. Warm‐night treatment reduced male lifespan, but had a positive impact on his reproduction and on offspring performance. Horizontal lines in the box plots represent the median, 25th, and 75th percentiles; whiskers indicate the values within 1.5 times the interquartile range and circles represent outliers

Against our prediction, exposure to warmer night conditions did not affect female lifespan (*p* = .5, Table S2). Female lifetime egg production was also not affected by the temperature treatment (*p* > .3 for all, Table S2), whereas the hatching success of the eggs produced was lower in females exposed to warm conditions at night (*χ* ^2^_1,29_ = 36.72, *p* < .0001; Table S2). On the contrary, warm‐night exposure did reduce male lifespan (*F*_1,74_ = 4.4, *p* = .04, Figure 4a, Table S2). Furthermore, females that mated with warm‐night males produced larger egg clutches (*F*_1,14_ = 6.5, *p* = .02, Figure 4b, Table S3). Furthermore, eggs sired by warm‐exposed males had higher hatching success (*χ* ^2^_1,15_ = 38.4, *p* < .0001, Figure 4c, Table S2). Generally, larger pupal mass (indicating also larger body size as an adult) increased male mating success (*χ* ^2^_1,72_ = 7.3, *p* = .007), the number of clutches he sired (*F*_1,16_ = 4.6, *p* = .05), offspring hatching success for both sexes (females: *χ* ^2^_1,29_ = 242, *p* < .0001, males: *χ* ^2^_1,15_ = 376.8, *p* < .0001), and male adult lifespan (*F*_1,74_ = 5.1, *p* = .02, Table S2).

## 4 DISCUSSION

Climate change has been unequivocally shown to cause changes in the phenology of living organisms (Menzel et al., 2006; Ovaskainen et al., 2013; Walther, 2010), and altering critical life history characteristics, such as the timing of reproduction (Spottiswoode et al., 2006). As temperatures have been shown to be rising faster during the night than during the day (Cox et al., 2020), we wanted to test the ability of a temperate‐zone insect to respond to warming night temperatures. Because the environmental conditions where organisms spend the initial and most sensitive stages of their lives are critical to individual performance, especially when those life stages are sessile, we wanted to assess whether warm conditions induce behavioral changes in maternal oviposition site choice. We expected females, who are generally known to prefer warm microhabitats for oviposition, to switch their preference toward more shaded sites after experiencing warm‐night conditions. This anticipatory behavioral switch may then spare their offspring from an increased drought risk. We were also interested in assessing whether warm‐night exposure impacted parent–offspring resource allocation. As warmer nights are known to increase adult metabolism (Niitepõld, 2010), we predicted it would also accelerate their pace of life (Pearl, 1928), and result in altered resource allocation patterns toward the offspring. To address these questions, we assessed first the general oviposition preference between dry and lush host plants occurring in open or canopy‐covered sites, respectively, resembling the habitat type preferred by the butterflies in normal climatic conditions (i.e., dry meadows) and one less preferred but granting better chances for host plants to endure throughout the summer (i.e., pasture margins or shaded areas within dry meadows). We then assessed offspring performance and linked it with the parents’ night thermal treatment and maternal oviposition preference behavior.

Work on the butterfly preference–performance hypothesis has often shown inconsistent results between the oviposition sites that are preferably chosen by females and the plants on which their offspring perform best (e.g., Griese et al., 2020; Ladner & Altizer, 2005; Rausher, 1979). This indicates that the choice for oviposition sites is dictated only partially by plant quality and that the environmental conditions may play a more important role in determining female site choice. The Glanville fritillary butterfly is no exception to this, and females within the Northern range boundaries have been recently shown to oviposit in microhabitats that are beneficial for the offspring performance on normal years, but that are likely to become too dry as climate warms, such as south‐facing slopes and sunny areas (Salgado et al., 2020). We found here that the exposure to warm‐night conditions had no influence on maternal oviposition site preference, as all butterflies, regardless of treatment, showed a marked predisposition to visit and stay in open areas rather than in canopy‐covered ones. Sunny and open areas further maximized butterfly reproductive activities such as matings and ovipositions. Similarly, recent work on *Pararge aegeria* has shown that egg laying almost invariably occurs in direct sunlight, even in shaded habitats, and independently of the temperature (Braem & Van Dyck, 2021). We further show that canopy‐covered areas were visited with lower frequency and may even have acted as traps where oviposition generally took longer. Hence, our results are in line with the general rule that, especially at high altitudes and latitudes, ectothermic organisms like insects depend strongly on sunlight for development and flight (Heinrich, 1993; Kammer & Heinrich, 1978).

We also found an oviposition preference for well‐watered plants and also in this case the choice was univocal, regardless of the female night thermal treatments. This suggests that the choice for host plant individuals is generally aimed at maximizing offspring survival (Salgado & Saastamoinen, 2019), whenever these lush plants occur in a sunny site where the females are more likely to fly actively. This is in line with literature showing that ovipositing females first choose a patch of suitable habitat and only then proceed with the choice of individual plants within that site (Friberg et al., 2008; Friberg & Wiklund, 2019; Wiklund & Friberg, 2008). On the other hand, it has also been shown that piercing–sucking herbivores are more abundant under intermittent water stress (Sconiers & Eubanks, 2017) and that drought favors the outbreak of some insects (Mattson & Haack, 1987). However, these patterns depend strongly on the insect diet, degree of specificity for the host plant, and degree of drought exposure.

We expected that our warm‐night treatment would increase the metabolism of these temperate‐zone butterflies (Dillon et al., 2010) and consequently impact their rate of living, which would then lead to lifespan reduction (as suggested by the rate of living and oxidative damage theories of aging Harman, 1956; Pearl, 1928) and changes in resource allocation patterns for reproduction. We found that the offspring of warm‐night exposed females suffered a ~10% reduction in hatching success, suggesting that the treatment had a negative impact on female resource allocation. It is possible, for example, that exposure to warmer conditions altered oogenesis by reducing eggs size (not measured here) as found in *Bicyclus anaynana* (Fischer et al., 2003). Alternatively, some other measures of egg viability, which may consequently impact hatching rate, may have been altered. Interestingly, however, female lifespan was not reduced by warm‐night exposure, nor was the number of eggs they laid.

Conversely, warm‐night exposure did reduce lifespan in males. Moreover, exposure to warm night increased male fitness and his offspring performance, as females that had mated with warm‐exposed males laid larger clutches, whose eggs also had a higher hatching success. This suggests a positive effect of warm‐night exposure on male resource allocation patterns. This change in reproductive strategy (i.e., increased investment in current reproduction rather than in future reproduction) by warm‐exposed males may be mediated by a higher investment in sperm quality, rather than in nuptial gifts. It is noteworthy, however, that while spermatophore content (Wedell & Karlsson, 2003; Wiklund et al., 1993) and/or the presence of additional male‐delivered nutrients are known to increase female fecundity in other Lepidoptera (Boggs & Gilbert, 1979), larger spermatophores in the Glanville fritillary butterfly do not increase paternity chances or male fertilization success (Duplouy et al., 2018). Another possibility is that the warm‐exposure at night impacted spermatophore content, resulting in larger egg clutches and higher hatching success. It is also possible that warm‐exposed males had a faster pace of life, reached their prime of life earlier, ultimately leading to higher reproductive success. Spermatophore size is known to increase with male age at first mating (Duplouy et al., 2018), suggesting that very young or slowly maturing males may be not be the best fathers. Positive effects of male maturity (i.e., increased age) on reproductive potential have been shown with bush crickets (Lehmann & Lehmann, 2009) and the butterfly *Bicyclus anynana* (Kehl et al., 2013). Finally, yet another possibility is that warm‐night males invested more in reproduction by mating longer with the females, which would then result in a higher hatching success of the eggs they sired. Unfortunately, mating duration was not tested here. Our results also suggest that male condition impacted female oviposition preference as the generally preferred host plat in the present experiment, *P. lanceolata*, was selected even more frequently by females that had mated with warm‐exposed males. *Plantago lanceolata* is more widespread in the Åland Islands (Nieminen et al., 2004), yet butterflies tend to show preference for *V. spicata* in sites where both host plants are present (Saastamoinen et al., 2013). As we do not know the underlying reason for the increased fitness benefit of mating with warm‐exposed males, it is difficult to speculate why female host plant preference was also modified. For example, increased investment in oviposition plant choice by females who mated with warm‐treated males may even reflect a females’ attempt to neutralize any potential negative effects inherited from fathers (i.e., “Compensation Hypothesis,” Gowaty et al., 2007). We are unaware of previous work showing a role of male condition biasing female oviposition choice, and hence, the mechanisms underlying this finding require further studies.

Night temperature is an important factor allowing diurnal ectothermic organisms to lower their metabolism and maximize their energy budget for the active hours of the day (Helfrich‐Förster, 2018). We show that higher temperatures, here experienced during the night time, reduced lifespan in males, while females in general were less affected by the treatment. Such sex‐dependent response may be explained by sexual size dimorphism, whereby males are consistently smaller than females (Allen et al., 2011). It is possible that the smaller size of males makes them more susceptible to the physical effects of warm temperatures. Small size is frequently linked with reduced fitness (Kingsolver & Huey, 2008), and we also show here that small males, regardless of treatment, have reduced lifespan, mating success, sire fewer clutches, and offspring with lower hatching rate. Moreover, the two sexes are evolutionarily wired differently, as males typically benefit from early emergence and primarily aim at getting mated, while females benefit from being more resilient and living longer to maximize the chances of laying all their eggs (Allen et al., 2011). These sex‐specific differences need to be carefully considered in ecological studies, also in regard to paternal effects, which are frequently overlooked (Crean et al., 2013).

Warm‐night temperatures have become a more than likely prospective under the climate change scenario, and organisms able to respond promptly will have better chances to endure in future. In the present work, we found that female Glanville fritillary butterflies at their Northern range margin showed no signs of changing their “usual” oviposition site selection strategies, even though preference for open and warm microclimatic conditions has been proved to be risky and cause the local populations to undergo severe bottlenecks under extremely warm and dry summers (van Bergen et al., 2020). Our results further highlight that climate change may differentially impact the two sexes and potentially influence sexual selection. If warmer summers became the rule, short‐lived males may for example foster an increase in male monogamy, with repercussions on the genetic composition of future generations. If this is combined with the bottlenecks caused by a risky oviposition site selection strategy, the genetic pool of the metapopulation could be severely reduced within few years. Our work adds on to an already long list of consequences of warming climate on the phenology of wild animal populations, and highlights the importance of the, yet often‐disregarded, paternal effects.

## CONFLICT OF INTERESTS

None declared.

## AUTHOR CONTRIBUTIONS

**Elena Rosa:** Conceptualization (equal); Formal analysis (lead); Investigation (lead); Methodology (lead); Visualization (lead); Writing‐original draft (equal). **Marjo Saastamoinen:** Conceptualization (equal); Funding acquisition (lead); Supervision (lead); Writing‐original draft (equal).

## ETHICAL APPROVAL

Insects and plants are not legally concerned by ethical regulations.

## Supporting information

Appendix S1‐S2

## Data Availability

Data associated with this experiment can be found at https://doi.org/10.5061/dryad.zcrjdfncs.
