# Supplementary material for: Warm‐night temperature alters paternal allocation strategy in a North temperate‐zone butterfly
Source: Ecol Evol. 2021 Nov 23;11(23):16514–23. doi: 10.1002/ece3.8120 (PMC8668742; doi:10.1002/ece3.8120)
Supplement: Supplementary file 1 — Appendix S1‐S2 [file ECE3-11-16514-s001.docx]

**Appendix S1**

**Table S1**: Statistical models for global oviposition preference (i.e. by clutch).

| **Response variable** | **Fixed effects** | ***Df*** | ***N*** | ***χ2*** | ***Pr(>χ2)*** |  |
| --- | --- | --- | --- | --- | --- | --- |
| ***V. spicata* vs *P. lanceolata*** | clutch rank | 1 | 95 | 0.47 | 0.49 |  |
|  | female night treatment | 1 | 95 | 0.69 | 0.41 |  |
|  | male night treatment | 1 | 95 | 7.90 | 0.00 | ** |
|  |  |  |  |  |  |  |
| **Lush vs dry** | clutch rank | 1 | 95 | 1.78 | 0.18 |  |
|  | female night  treatment | 1 | 95 | 0.07 | 0.79 |  |
|  | male night  treatment | 1 | 95 | 0.11 | 0.74 |  |
|  |  |  |  |  |  |  |
| **Open vs canopy** | clutch rank | 1 | 95 | 0.77 | 0.37 |  |
|  | female night  treatment | 1 | 95 | 0.85 | 0.36 |  |
|  | male night treatment | 1 | 95 | 2.64 | 0.10 |  |
|  |  |  |  |  |  |  |

**Table S2**: Statistical models for fitness traits

| **Response variable** | **Fixed effects** | ***Df*** | ***N*** | ***χ2*** | ***Pr(>χ2)*** |  |
| --- | --- | --- | --- | --- | --- | --- |
| **Mated (♀)** | female night treatment | 1 | 81 | 0.01 | 0.92 |  |
|  | pupal mass  f. night treat:p. mass | 1  1 | 81  81 | 0.90  0.003 | 0.34  0.96 |  |
|  |  |  |  |  |  |  |
| **Mated (♂)** | male night treatment | 1 | 72 | 0.57 | 0.45 |  |
|  | pupal mass  m. night treat:p. mass | 1  1 | 72  72 | 7.31  0.097 | 0.007  0.76 | ** |
|  |  |  |  |  |  |  |
| **Oviposited (♀)** | female night treatment | 1 | 61 | 0.00 | 0.99 |  |
|  | pupal mass  f. night treat:p. mass | 1  1 | 61  61 | 1.37  0.76 | 0.24  0.38 |  |
|  |  |  |  |  |  |  |
| **Sired eggs (♂)** | male night treatment | 1 | 39 | 0.07 | 0.79 |  |
|  | pupal mass  m. night treat:p. mass | 1  1 | 39  39 | 0.56  1.18 | 0.46  0.28 |  |
|  |  |  |  |  |  |  |
|  |  | ***Df*** | ***Den(df)*** | ***F*** | ***Pr(>F)*** |  |
| **Lifetime clutches (♀)** | female night treatment | 1 | 30 | 0.17 | 0.68 |  |
|  | pupal mass  f. night treat:p. mass | 1  1 | 30  30 | 0.12  0.12 | 0.73  0.74 |  |
|  |  |  |  |  |  |  |
| **Lifetime clutches (♂)** | male night treatment | 1 | 16 | 1.01 | 0.32 |  |
|  | pupal mass  m. night treat:p. mass | 1  1 | 16  16 | 4.64  0.68 | 0.047  0.42 | * |
|  |  |  |  |  |  |  |
| **Lifetime eggs (♀)** | female night treatment | 1 | 30 | 1.07 | 0.31 |  |
|  | pupal mass  f. night treat:p. mass | 1  1 | 30  30 | 2.39  0.83 | 0.13  0.37 |  |
|  |  |  |  |  |  |  |
| **Lifetime eggs (♂)** | male night treatment | 1 | 16 | 0.18 | 0.67 |  |
|  | pupal mass  m. night treat:p. mass | 1  1 | 16  16 | 0.79  0.13 | 0.39  0.72 |  |
|  |  |  |  |  |  |  |
|  |  | ***Df*** | ***N*** | ***χ2*** | ***Pr(>χ2)*** |  |
| **Lifetime hatching (♀)** | female night treatment | 1 | 29 | 36.72 | <0.0001 | *** |
|  | pupal mass  f. night treat:p. mass | 1  1 | 29  29 | 242.00  205.58 | <0.0001  <0.0001 | ***  *** |
|  |  |  |  |  |  |  |
| **Lifetime hatching (♂)** | male night treatment | 1 | 15 | 38.42 | <0.0001 | *** |
|  | pupal mass  m. night treat:p. mass | 1  1 | 15  15 | 376.84  40.94 | <0.0001  <0.0001 | ***  *** |
|  |  |  |  |  |  |  |
| **Lifespan (♀)** | female night treatment | 1 | 82 | 0.46 | 0.50 |  |
|  | pupal mass  f. night treat:p. mass | 1  1 | 82  82 | 0.41  0.01 | 0.52  0.92 |  |
|  |  |  |  |  |  |  |
| **Lifespan (♂)** | male night treatment | 1 | 74 | 4.40 | 0.036 | * |
|  | pupal mass  m. night treat:p. mass | 1  1 | 74  74 | 5.14  0.06 | 0.023  0.81 | * |
|  |  |  |  |  |  |  |

**Table S3**: Statistical models for other measures relative to individual ovipositions. Model selection was done based on the lowest AIC value with ΔAIC>4 as threshold. When ΔAIC was lower than 4, we performed model averaging. Parameter estimates are reported with F-statistics when the best model had a ΔAIC higher than 4 and with Z-statistics when model averaging was performed.

| **Response variable** |  |  | ***Df*** | ***Den(df)*** | ***F*** | ***Pr(>F)*** |  |
| --- | --- | --- | --- | --- | --- | --- | --- |
| **Clutch size** | female night treat. |  | 1 | 11.66 | 2.63 | 0.13 |  |
|  | clutch rank |  | 1 | 61.39 | 5.88 | 0.018 | * |
|  | plant type |  | 3 | 56.99 | 6.11 | 0.001 | ** |
|  | oviposition site |  | 1 | 65.21 | 0.63 | 0.43 |  |
|  | male night treat. |  | 1 | 13.47 | 6.46 | 0.024 | * |
|  |  |  |  |  |  |  |  |
|  |  | ***Estimate*** | ***SE*** | ***Adjusted SE*** | ***z value*** | ***Pr(>\|z\|)*** |  |
| **Oviposition time** | (Intercept) | 13.04 | 0.25 | 0.26 | 50.71 | <0.0001 | *** |
|  | plant type (*P.l.* dry) | -0.17 | 0.32 | 0.33 | 0.51 | 0.61 |  |
|  | plant type (*V.s.* lush) | -0.03 | 0.19 | 0.19 | 0.18 | 0.86 |  |
|  | plant type (*V.s.* dry) | 0.20 | 0.38 | 0.39 | 0.52 | 0.60 |  |
|  | ovip. site (canopy) | -0.00 | 0.23 | 0.24 | 0.02 | 0.99 |  |
|  | female treat. (warm n.) | 0.06 | 0.18 | 0.18 | 0.36 | 0.72 |  |
|  | clutch rank | -0.02 | 0.05 | 0.05 | 0.41 | 0.68 |  |
|  | male treat. (warm n.) | 0.01 | 0.13 | 0.13 | 0.11 | 0.91 |  |
|  |  |  |  |  |  |  |  |
| **Oviposition duration (min)** | (Intercept) | 34.78 | 8.85 | 9.02 | 3.86 | 0.0001 | *** |
|  | clutch rank | -0.29 | 1.02 | 1.03 | 0.29 | 0.78 |  |
|  | female treat. (warm n.) | -5.10 | 8.13 | 8.28 | 0.62 | 0.54 |  |
|  | male treat. (warm n.) | 0.53 | 8.17 | 8.33 | 0.06 | 0.95 |  |
|  | plant type (*P.l.* dry) | -1.07 | 7.74 | 7.88 | 0.14 | 0.89 |  |
|  | plant type (*V.s.* lush) | 10.06 | 7.17 | 7.31 | 1.38 | 0.17 |  |
|  | plant type (*V.s.* dry) | 6.75 | 9.21 | 9.39 | 0.72 | 0.47 |  |
|  | ovip. site (canopy) | 20.28 | 9.46 | 9.63 | 2.11 | 0.035 | * |
|  |  |  |  |  |  |  |  |

**Appendix S2**

**Table S4:** Release and recollection logs of each butterfly used in the experiment. "Recollection success" is based on the number of times a butterfly was recollected divided by the total number of recollection days during its lifespan. "Treatment success" is based on the total number of nights a butterfly spent indoors in the assigned night treatment (control/warm) divided by the total number of nights it could have spent indoors (i.e. if recollection success was always 100%).

| **ID** | **Night**  **treatment** | **Sex** | **Times recollected** | **Recollection**  **days tot.** | **Recollection success (%)** | **Nights in** | **Nights in tot.** | **Treatment success (%)** |
| --- | --- | --- | --- | --- | --- | --- | --- | --- |
| 1 | control | ♂ | 15 | 17 | **0.88** | 17 | 20 | **0.85** |
| 2 | warm | ♂ | 6 | 7 | **0.86** | 7 | 9 | **0.78** |
| 3 | control | ♂ | 10 | 10 | **1.00** | 11 | 12 | **0.92** |
| 4 | warm | ♂ | 3 | 4 | **0.75** | 4 | 6 | **0.67** |
| 5 | control | ♀ | 4 | 6 | **0.67** | 6 | 7 | **0.86** |
| 6 | warm | ♀ | 3 | 5 | **0.60** | 5 | 6 | **0.83** |
| 7 | control | ♂ | 6 | 8 | **0.75** | 7 | 9 | **0.78** |
| 8 | warm | ♂ | 8 | 14 | **0.57** | 9 | 17 | **0.53** |
| 9 | control | ♂ | 0 | 0 | **0.00** | 1 | 1 | **1.00** |
| 10 | warm | ♂ | 5 | 6 | **0.83** | 7 | 7 | **1.00** |
| 11 | control | ♀ | 14 | 17 | **0.82** | 18 | 20 | **0.90** |
| 12 | warm | ♂ | 6 | 11 | **0.55** | 8 | 12 | **0.67** |
| 13 | control | ♂ | 11 | 12 | **0.92** | 13 | 14 | **0.93** |
| 14 | warm | ♀ | 6 | 8 | **0.75** | 8 | 9 | **0.89** |
| 15 | control | ♂ | 5 | 8 | **0.63** | 6 | 9 | **0.67** |
| 16 | warm | ♀ | 15 | 18 | **0.83** | 17 | 21 | **0.81** |
| 17 | control | ♂ | 5 | 8 | **0.63** | 7 | 9 | **0.78** |
| 18 | warm | ♂ | 2 | 3 | **0.67** | 4 | 5 | **0.80** |
| 19 | control | ♂ | 10 | 11 | **0.91** | 12 | 12 | **1.00** |
| 20 | warm | ♂ | 3 | 4 | **0.75** | 5 | 6 | **0.83** |
| 21 | control | ♂ | 8 | 9 | **0.89** | 9 | 11 | **0.82** |
| 22 | warm | ♂ | 4 | 6 | **0.67** | 5 | 8 | **0.63** |
| 23 | control | ♀ | 4 | 5 | **0.80** | 5 | 7 | **0.71** |
| 24 | warm | ♂ | 5 | 5 | **1.00** | 7 | 7 | **1.00** |
| 25 | control | ♀ | 2 | 4 | **0.50** | 3 | 6 | **0.50** |
| 26 | warm | ♀ | 6 | 9 | **0.67** | 8 | 10 | **0.80** |
| 27 | control | ♂ | 8 | 9 | **0.89** | 10 | 11 | **0.91** |
| 28 | warm | ♀ | 7 | 8 | **0.88** | 9 | 10 | **0.90** |
| 29 | control | ♀ | 3 | 4 | **0.75** | 5 | 5 | **1.00** |
| 30 | warm | ♀ | 9 | 11 | **0.82** | 11 | 13 | **0.85** |
| 31 | control | ♂ | 6 | 7 | **0.86** | 8 | 8 | **1.00** |
| 32 | warm | ♂ | 7 | 8 | **0.88** | 9 | 10 | **0.90** |
| 33 | control | ♀ | 9 | 16 | **0.56** | 10 | 19 | **0.53** |
| 34 | warm | ♂ | 12 | 14 | **0.86** | 15 | 18 | **0.83** |
| 35 | control | ♂ | 6 | 9 | **0.67** | 8 | 10 | **0.80** |
| 36 | warm | ♂ | 8 | 11 | **0.73** | 10 | 12 | **0.83** |
| 37 | control | ♀ | 12 | 17 | **0.71** | 16 | 21 | **0.76** |
| 38 | warm | ♂ | 4 | 4 | **1.00** | 6 | 6 | **1.00** |
| 39 | control | ♂ | 6 | 8 | **0.75** | 7 | 9 | **0.78** |
| 40 | warm | ♂ | 4 | 5 | **0.80** | 6 | 6 | **1.00** |
| 41 | control | ♂ | 3 | 3 | **1.00** | 4 | 5 | **0.80** |
| 42 | warm | ♂ | 5 | 7 | **0.71** | 6 | 8 | **0.75** |
| 43 | control | ♂ | 12 | 13 | **0.92** | 14 | 15 | **0.93** |
| 44 | warm | ♂ | 5 | 6 | **0.83** | 7 | 8 | **0.88** |
| 45 | control | ♂ | 4 | 5 | **0.80** | 6 | 6 | **1.00** |
| 46 | warm | ♀ | 14 | 16 | **0.88** | 18 | 19 | **0.95** |
| 47 | control | ♀ | 6 | 7 | **0.86** | 8 | 9 | **0.89** |
| 48 | warm | ♂ | 9 | 11 | **0.82** | 11 | 12 | **0.92** |
| 49 | control | ♂ | 7 | 9 | **0.78** | 9 | 11 | **0.82** |
| 50 | warm | ♀ | 9 | 11 | **0.82** | 10 | 12 | **0.83** |
| 51 | control | ♀ | 6 | 6 | **1.00** | 8 | 8 | **1.00** |
| 52 | warm | ♀ | 10 | 14 | **0.71** | 14 | 17 | **0.82** |
| 53 | control | ♂ | 12 | 13 | **0.92** | 14 | 15 | **0.93** |
| 54 | warm | ♂ | 6 | 10 | **0.60** | 8 | 11 | **0.73** |
| 55 | control | ♀ | 6 | 7 | **0.86** | 8 | 8 | **1.00** |
| 56 | warm | ♀ | 6 | 6 | **1.00** | 8 | 8 | **1.00** |
| 57 | control | ♀ | 3 | 6 | **0.50** | 5 | 7 | **0.71** |
| 58 | warm | ♂ | 3 | 4 | **0.75** | 5 | 5 | **1.00** |
| 59 | control | ♀ | 2 | 5 | **0.40** | 4 | 6 | **0.67** |
| 60 | warm | ♀ | 5 | 8 | **0.63** | 6 | 9 | **0.67** |
| 61 | control | ♀ | 1 | 2 | **0.50** | 3 | 3 | **1.00** |
| 62 | warm | ♂ | 6 | 6 | **1.00** | 8 | 8 | **1.00** |
| 63 | control | ♀ | 0 | 1 | **0.00** | 1 | 1 | **1.00** |
| 64 | warm | ♀ | 4 | 4 | **1.00** | 6 | 6 | **1.00** |
| 65 | control | ♂ | 11 | 13 | **0.85** | 15 | 16 | **0.94** |
| 66 | warm | ♀ | 4 | 6 | **0.67** | 6 | 7 | **0.86** |
| 67 | control | ♀ | 11 | 13 | **0.85** | 15 | 16 | **0.94** |
| 68 | warm | ♀ | 5 | 6 | **0.83** | 7 | 7 | **1.00** |
| 69 | control | ♂ | 12 | 13 | **0.92** | 16 | 16 | **1.00** |
| 70 | warm | ♂ | 5 | 5 | **1.00** | 7 | 7 | **1.00** |
| 71 | control | ♂ | 9 | 10 | **0.90** | 10 | 12 | **0.83** |
| 72 | warm | ♀ | 4 | 10 | **0.40** | 6 | 12 | **0.50** |
| 73 | control | ♂ | 10 | 11 | **0.91** | 12 | 13 | **0.92** |
| 74 | warm | ♀ | 13 | 16 | **0.81** | 17 | 19 | **0.89** |
| 75 | control | ♀ | 7 | 9 | **0.78** | 9 | 11 | **0.82** |
| 76 | warm | ♀ | 7 | 7 | **1.00** | 8 | 9 | **0.89** |
| 77 | control | ♀ | 7 | 9 | **0.78** | 8 | 10 | **0.80** |
| 78 | warm | ♂ | 1 | 2 | **0.50** | 3 | 3 | **1.00** |
| 79 | control | ♀ | 3 | 8 | **0.38** | 4 | 9 | **0.44** |
| 80 | warm | ♀ | 15 | 16 | **0.94** | 19 | 19 | **1.00** |
| 81 | control | ♂ | 2 | 3 | **0.67** | 4 | 4 | **1.00** |
| 82 | warm | ♀ | 6 | 8 | **0.75** | 7 | 9 | **0.78** |
| 83 | control | ♂ | 2 | 4 | **0.50** | 4 | 5 | **0.80** |
| 84 | warm | ♀ | 7 | 8 | **0.88** | 9 | 9 | **1.00** |
| 85 | control | ♂ | 8 | 10 | **0.80** | 10 | 11 | **0.91** |
| 86 | warm | ♀ | 4 | 6 | **0.67** | 6 | 7 | **0.86** |
| 87 | control | ♀ | 9 | 10 | **0.90** | 11 | 11 | **1.00** |
| 88 | warm | ♂ | 5 | 8 | **0.63** | 6 | 10 | **0.60** |
| 89 | control | ♂ | 5 | 7 | **0.71** | 7 | 8 | **0.88** |
| 90 | warm | ♀ | 2 | 3 | **0.67** | 4 | 4 | **1.00** |
| 91 | control | ♀ | 8 | 8 | **1.00** | 10 | 10 | **1.00** |
| 92 | warm | ♀ | 2 | 5 | **0.40** | 4 | 6 | **0.67** |
| 93 | control | ♀ | 11 | 13 | **0.85** | 15 | 16 | **0.94** |
| 94 | warm | ♂ | 2 | 3 | **0.67** | 4 | 5 | **0.80** |
| 95 | control | ♀ | 7 | 10 | **0.70** | 9 | 12 | **0.75** |
| 96 | warm | ♀ | 7 | 13 | **0.54** | 9 | 16 | **0.56** |
| 97 | control | ♀ | 6 | 9 | **0.67** | 8 | 10 | **0.80** |
| 98 | warm | ♂ | 7 | 7 | **1.00** | 9 | 9 | **1.00** |
| 99 | control | ♂ | 8 | 8 | **1.00** | 10 | 10 | **1.00** |
| 100 | warm | ♂ | 1 | 2 | **0.50** | 3 | 3 | **1.00** |
| 101 | control | ♂ | 5 | 6 | **0.83** | 6 | 8 | **0.75** |
| 102 | warm | ♂ | 14 | 15 | **0.93** | 16 | 19 | **0.84** |
| 103 | control | ♀ | 4 | 5 | **0.80** | 6 | 6 | **1.00** |
| 104 | warm | ♂ | 9 | 10 | **0.90** | 11 | 11 | **1.00** |
| 105 | control | ♂ | 11 | 11 | **1.00** | 13 | 13 | **1.00** |
| 106 | warm | ♀ | 10 | 11 | **0.91** | 12 | 13 | **0.92** |
| 107 | control | ♂ | 5 | 5 | **1.00** | 6 | 7 | **0.86** |
| 108 | warm | ♀ | 12 | 14 | **0.86** | 16 | 17 | **0.94** |
| 109 | control | ♀ | 16 | 17 | **0.94** | 19 | 20 | **0.95** |
| 110 | warm | ♀ | 7 | 8 | **0.88** | 9 | 9 | **1.00** |
| 111 | control | ♀ | 11 | 13 | **0.85** | 14 | 16 | **0.88** |
| 112 | warm | ♂ | 4 | 6 | **0.67** | 6 | 7 | **0.86** |
| 113 | control | ♀ | 0 | 0 | **0.00** | 1 | 1 | **1.00** |
| 114 | warm | ♀ | 10 | 12 | **0.83** | 13 | 14 | **0.93** |
| 115 | control | ♀ | 3 | 5 | **0.60** | 4 | 5 | **0.80** |
| 116 | warm | ♀ | 3 | 5 | **0.60** | 4 | 6 | **0.67** |
| 117 | control | ♀ | 6 | 6 | **1.00** | 7 | 7 | **1.00** |
| 118 | warm | ♀ | 8 | 9 | **0.89** | 9 | 9 | **1.00** |
| 119 | control | ♀ | 7 | 7 | **1.00** | 8 | 8 | **1.00** |
| 120 | warm | ♀ | 12 | 12 | **1.00** | 15 | 15 | **1.00** |
| 121 | control | ♂ | 8 | 9 | **0.89** | 9 | 10 | **0.90** |
| 122 | warm | ♂ | 1 | 4 | **0.25** | 2 | 4 | **0.50** |
| 123 | control | ♀ | 7 | 8 | **0.88** | 8 | 9 | **0.89** |
| 124 | warm | ♀ | 7 | 7 | **1.00** | 8 | 8 | **1.00** |
| 125 | control | ♀ | 11 | 11 | **1.00** | 12 | 12 | **1.00** |
| 126 | warm | ♂ | 10 | 11 | **0.91** | 11 | 12 | **0.92** |
| 127 | control | ♂ | 7 | 8 | **0.88** | 8 | 9 | **0.89** |
| 128 | warm | ♂ | 4 | 4 | **1.00** | 5 | 5 | **1.00** |
| 129 | control | ♂ | 13 | 14 | **0.93** | 16 | 17 | **0.94** |
| 130 | warm | ♀ | 1 | 3 | **0.33** | 2 | 3 | **0.67** |
| 131 | control | ♀ | 5 | 6 | **0.83** | 6 | 6 | **1.00** |
| 132 | warm | ♂ | 2 | 2 | **1.00** | 3 | 3 | **1.00** |
| 133 | control | ♀ | 1 | 2 | **0.50** | 2 | 2 | **1.00** |
| 134 | warm | ♀ | 2 | 7 | **0.29** | 3 | 7 | **0.43** |
| 135 | control | ♂ | 2 | 3 | **0.67** | 3 | 3 | **1.00** |
| 136 | warm | ♂ | 3 | 3 | **1.00** | 4 | 4 | **1.00** |
| 137 | control | ♂ | 6 | 8 | **0.75** | 7 | 8 | **0.88** |
| 138 | warm | ♀ | 2 | 3 | **0.67** | 3 | 3 | **1.00** |
| 139 | control | ♀ | 3 | 3 | **1.00** | 4 | 4 | **1.00** |
| 140 | warm | ♀ | 4 | 6 | **0.67** | 5 | 6 | **0.83** |
| 141 | control | ♀ | 0 | 1 | **0.00** | 1 | 1 | **1.00** |
| 142 | warm | ♀ | 3 | 3 | **1.00** | 4 | 4 | **1.00** |
| 143 | control | ♂ | 0 | 7 | **0.00** | 1 | 7 | **0.14** |
| 144 | warm | ♀ | 2 | 7 | **0.29** | 3 | 8 | **0.38** |
| 145 | control | ♂ | 7 | 8 | **0.88** | 8 | 9 | **0.89** |
| 146 | warm | ♂ | 7 | 7 | **1.00** | 8 | 8 | **1.00** |
| 147 | control | ♂ | 5 | 5 | **1.00** | 6 | 6 | **1.00** |
| 148 | warm | ♀ | 5 | 8 | **0.63** | 6 | 8 | **0.75** |
| 149 | control | ♀ | 11 | 13 | **0.85** | 14 | 15 | **0.93** |
| 150 | warm | ♀ | 6 | 6 | **1.00** | 7 | 7 | **1.00** |
| 151 | control | ♂ | 4 | 5 | **0.80** | 5 | 5 | **1.00** |
| 152 | warm | ♀ | 8 | 11 | **0.73** | 11 | 13 | **0.85** |
| 153 | control | ♀ | 6 | 7 | **0.86** | 7 | 8 | **0.88** |
| 154 | warm | ♀ | 5 | 6 | **0.83** | 6 | 6 | **1.00** |
| 155 | control | ♂ | 14 | 15 | **0.93** | 17 | 17 | **1.00** |
| 156 | warm | ♀ | 10 | 11 | **0.91** | 13 | 13 | **1.00** |
| 157 | control | ♂ | 7 | 9 | **0.78** | 8 | 10 | **0.80** |
| 158 | warm | ♂ | 12 | 13 | **0.92** | 15 | 15 | **1.00** |
| 159 | control | ♀ | 11 | 11 | **1.00** | 14 | 14 | **1.00** |
| 160 | warm | ♀ | 11 | 13 | **0.85** | 14 | 16 | **0.88** |
| 161 | control | ♀ | 10 | 13 | **0.77** | 13 | 15 | **0.87** |
| 162 | warm | ♀ | 3 | 5 | **0.60** | 4 | 5 | **0.80** |
| 163 | control | ♀ | 6 | 6 | **1.00** | 7 | 7 | **1.00** |
|  |  |  | **Average recollection success (%):** | | **0.77** | **Average treatment success (%):** | | **0.89** |
